# Implications of the COVID-19 Pandemic for the Development and Update of Clinical Practice Guidelines: Viewpoint

**DOI:** 10.2196/20064

**Published:** 2020-12-29

**Authors:** Theresa Steeb, Markus Follmann, Ralf Matthias Hagen, Carola Berking, Markus Vincent Heppt

**Affiliations:** 1 Department of Dermatology Universitätsklinikum Erlangen Friedrich-Alexander-University Erlangen-Nürnberg Erlangen Germany; 2 German Cancer Society Berlin Germany; 3 Department of Microbiology and Hospital Hygiene Bundeswehr Central Hospital Koblenz Germany

**Keywords:** practice guideline, consensus development conference, guideline, videoconferencing, clinical practice, COVID-19, pandemic, public health, policy, decision making, online conference

## Abstract

Following the rapid spread of a new type of coronavirus (SARS-CoV-2), nearly all countries have introduced temporary restrictions affecting daily life, with “social distancing” as a key intervention for slowing the spread of the virus. Despite the pandemic, the development or actualization of medical guidelines, especially in the rapidly changing field of oncology, needs to be continued to provide up-to-date evidence- and consensus-based recommendations for shared decision making and maintaining the treatment quality for patients. In this viewpoint, we describe the potential strengths and limitations of online conferences for medical guideline development. This viewpoint will assist guideline developers in evaluating whether online conferences are an appropriate tool for their guideline conference and audience.

The world currently faces an outbreak of a new type of coronavirus (SARS-CoV-2), which began spreading in China at the end of 2019 and has now led to a worldwide pandemic [1]. The syndrome caused by SARS-CoV-2 (COVID-19) manifests clinically diversely, with some patients having an asymptomatic disease course while others suffer from pneumonia or life-threatening acute respiratory distress syndrome, which may lead to multisystem organ failure or death [2,3]. Since health care systems worldwide struggle with an unexpected large number of cases that need to be hospitalized, this pandemic represents a global public health threat of international concern [4].

Following the rapid spread of the virus, nearly all countries have introduced temporary restrictions affecting daily life, with “social distancing” as a key intervention for slowing the spread of the virus [5]. Besides these strategies, many companies have introduced or expanded home office–based work, which lead to a tremendous increase in the use of video web conference platforms such as *Skype for Business* (Microsoft Corporation), *Zoom* (Zoom Video Communications), *Google Hangouts* (Google LLC), *Adobe Connect* (Adobe Systems), or *GoToMeeting* (LogMeIn). A web conference or online meeting is a “virtual” meeting organized and conducted over the internet between participants in different locations. In a web conference, the desktop of the meeting moderator replaces the real conference table. All participants can follow what is happening on the moderator’s desktop in a window on their screen, such as holding a presentation or editing a text document. During the web conference the moderator’s role can be flexibly switched between the participants.

Until now, it remains unclear for how long temporary restrictions will be sustained. Despite the pandemic, the development or actualization of medical guidelines especially in the rapidly changing field of oncology needs to be continued to provide up-to-date evidence- and consensus-based recommendations for shared decision making and maintaining the treatment quality for patients. Furthermore, the COVID-19 pandemic itself is likely to affect specific recommendations of guidelines. This explains the urgent need to continue working on current guidelines.

Online conferences may help to replace face-to-face consensus meetings and ensure guideline development in real time while the pandemic is still ongoing. Face-to-face conferences require considerable personnel, financial, and time resources. In contrast, web-based conferences have the potential to reduce time efforts and cut costs, as business travels are unnecessary (Figure 1). The reduction of business travels may also contribute to a reduction in CO_2_ emissions, and therefore, online conferences may contribute to climate protection. Besides, collaboration is independent of location and, hence, strengthens international and interdisciplinary guideline work. Web-based conferences are easy to organize and may be scheduled spontaneously. This makes working in teams particularly efficient and may increase the productivity. Additionally, previous surveys showed that online consensus conferences combined with a telephone conference are a feasible and acceptable approach among guideline developers and participants, especially for participants with prior experience with consensus conferences [6,7]. However, technical issues like poor video quality, frozen screens, or choppy sound snippets may hinder guideline developers from realizing online conferences. In a survey among participants for the guideline development of actinic keratosis, the majority reported no technical problems with the participation in the online conference, whereas the possibility for discussion was rated to be possibly inferior to traditional face-to-face conferences [7]. Besides this, further limitations include miscommunication due to poor video quality, sometimes complicated and unintuitive operation of the software, and concerns regarding data security. Another disadvantage includes the lack of social interaction in online meetings as well as the lack of possibilities for networking. A lot of contacts, ideas, and opportunities arise from social interaction before and after meetings, particularly in small group face-to-face communication. In addition, young researchers could miss out on introducing themselves to potential mentors. Furthermore, the communication is only limited to one person at a time, and there is little room for criticism. Besides, difficulties in capturing mood and dynamics of the audience may remain elusive.

In summary, online conferences represent an important and precious tool to advance guideline development, especially during the COVID-19 pandemic but also for the future in the era of digitization. Nevertheless, to counteract possible disadvantages of web conferences, a moderator should be chosen to guide through the meeting and to warrant formal consensus finding processes. Besides, aims of the meeting, technical requirements, and rules for discussion should be defined and communicated in advance. Additionally, software of different providers should be compared to find the most suitable one. Future efforts should be undertaken to establish a framework for successful online conferences for guideline development [8].

**Figure 1:**
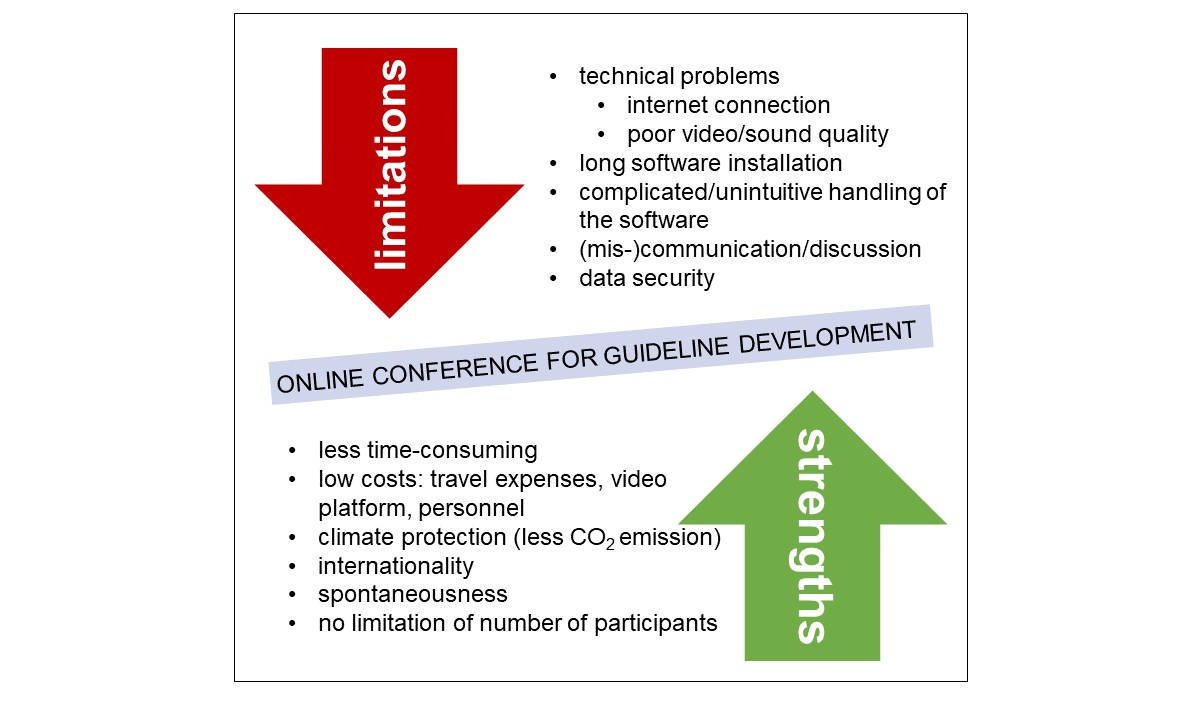
Strengths and limitations of online conferences for medical guideline development.
